# Phosphatidylinositol 4-kinase IIIβ (PI4KB) forms highly flexible heterocomplexes that include ACBD3, 14-3-3, and Rab11 proteins

**DOI:** 10.1038/s41598-018-37158-6

**Published:** 2019-01-24

**Authors:** Dominika Chalupska, Bartosz Różycki, Jana Humpolickova, Lenka Faltova, Martin Klima, Evzen Boura

**Affiliations:** 1 Institute of Organic Chemistry and Biochemistry of the Czech Academy of Sciences, Flemingovo nam. 2., Prague, Czech Republic; 2 Institute of Physics, Polish Academy of Sciences, Al. Lotnikow 32/46, 02-668, Warsaw, Poland; 3 Laboratory of Biomolecular Research, Department of Biology and Chemistry, Paul Scherrer Institute, 5232 Villigen, PSI Switzerland

## Abstract

Phosphatidylinositol 4-kinase IIIβ (PI4KB) is a key enzyme of the Golgi system because it produces its lipid hallmark - the phosphatidylinositol 4-phosphate (PI4P). It is recruited to Golgi by the Golgi resident ACBD3 protein, regulated by 14-3-3 proteins and it also serves as an adaptor because it recruits the small GTPase Rab11. Here, we analyzed the protein complexes formed by PI4KB *in vitro* using small angle x-ray scattering (SAXS) and we discovered that these protein complexes are highly flexible. The 14-3-3:PI4KB:Rab11 protein complex has 2:1:1 stoichiometry and its different conformations are rather compact, however, the ACBD3:PI4KB protein complex has both, very compact and very extended conformations. Furthermore, *in vitro* reconstitution revealed that the membrane is necessary for the formation of ACBD3:PI4KB:Rab11 protein complex at physiological (nanomolar) concentrations.

## Introduction

Phosphatidylinositol 4-phosphate (PI4P) is a key lipid for the identity of the Golgi and trans-Golgi network (TGN) and also serves as a precursor of phosphatidylinositol 4,5-bisphosphate (PI(4,5)P_2_), phosphatidylinositol 3,4-bisphosphate (PI(3,4)P_2_) and phosphatidylinositol 3,4,5-trisphosphate (PI(3,4,5)P_3_) and thus also functions as a signaling molecule^1,2^. The Golgi pool is in humans produced by the phosphatidylinositol 4-kinase IIIβ (PI4KB) and the phosphatidylinositol 4-kinase IIα (PI4K2A)^3–5^. Both PI4KB and PI4K2A are tightly regulated in the cell; they phosphorylate membranes and are activated by membrane recruitment. PI4KB is recruited to the membrane by the Golgi resident ACBD3 (Acyl-CoA-binding domain-containing) protein^6,7^ while the PI4K2A is palmitoylated and thus stably associated with the membrane upon palmitoylation^8,9^. PI4KB was also reported to be regulated and to regulate several proteins besides the above mentioned ACBD3 which includes 14-3-3 proteins^10^, the small GTPase Rab11^11^, neuronal calcium sensor-1^12^ (NCS-1 or frequenin in yeast) and the recently described C10orf76 of unknown function^13^. Importantly, PI4KB was recently recognized as an essential host factor for the replication of many +RNA viruses including Polio virus^14^, Hepatitis C virus^15^, Coxsackie virus^16^ and the human Aichi virus^6^ which sparked the interest of scientific community into PI4Ks and inevitably led us to solve their crystal structures^17–19^ which in turn helped the discovery of highly potent and selective PI4KB inhibitors that can be used as antivirals^20–22^ or as fluorescent tools^23^.

14-3-3 proteins are known to regulate hundreds of proteins in phosphorylation dependent manner^24^ including regulators of G-protein signaling^25^, transcription factors such as FOXO^26,27^ and multiple other enzymes. However, despite their importance only very few crystal structures (serotonin N-acetyltransferase, florigen Hd3a, small heat shock protein HSPB6, and the yeast neutral trehalase Nth1) of the full-length 14-3-3 protein complexes were solved^28–31^. 14-3-3 protein was also reported to increase the enzymatic activity of PI4KB in cells^10^, however, the structural analysis did not suggest any mechanism of 14-3-3 mediated activation of PI4KB and, congruently, no 14-3-3 dependent activation of PI4KB was observed using pure recombinant proteins *in vitro*^32^ suggesting a more complex mechanism of 14-3-3 dependent activation of PI4KB in cells.

**Figure 1:**
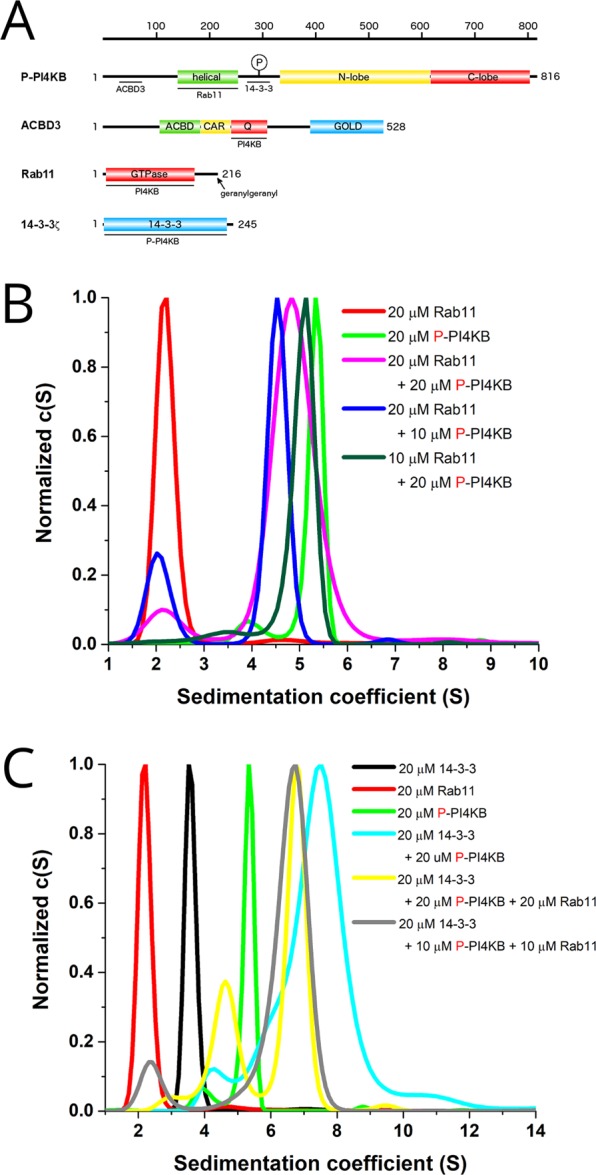
14-3-3, PI4KB and Rab11 form complex of 2:1:1 stoichiometry. (**A**) Schematic representation of the domain composition of the proteins used in the study. PI4KB is composed of the N-terminal region, helical domain, and kinase domain which can be divided into N- and C-terminal lobes. Phosphorylation site at S294 is indicated. ACBD3 contains the acyl-CoA binding domain (ACBD), charged amino acids region (CAR), glutamine rich region (Q), and Golgi dynamics domain (GOLD). Rab11 is composed of the N-terminal GTP-binding domain and disordered C-terminus which can be geranylgeranylated at C212 and C213 as indicated. (**B**,**C**) c (S) distribution profiles revealing that P-PI4KB:Rab11 complex is preferentially formed with 1:1 stoichiometry (**B**) and 14-3-3:P-PI4KB:Rab11complex with 2:1:1 stoichiometry (**C**). Sedimentation coefficients of all complexes analyzed by AUC are listed in SI Table 1.

On the other hand, regulation of PI4KB by the ACBD3 protein is rather well understood^7,33^. ACBD3 protein is Golgi localized and serves as a signaling hub^34^. It is composed of several domains (ACBD, CAR, Q, and the GOLD domains) connected by long intrinsically disordered linkers (Fig. 1A). The GOLD domain anchors ACBD3 to the Golgi via its interaction with giantin^35^ while the Q domain serves to bind the very N-terminal helix of PI4KB bringing it to close vicinity of the membrane and thus activating it^7^. Notably, viral 3A proteins bind the GOLD domain and literally pin it down to target membrane^36^ in order to recruit PI4KB to viral replication sites^37–39^.

Rab11 is a small GTPase that is membrane-localized because its C-terminus is geranylgeranylated, which is typical for this family of small GTPases^40,41^. Its interaction with PI4KB was structurally characterized and revealed that PI4KB does not affect neither switch I nor switch II region of Rab11^17^ and most like just serves to recruit Rab11 and its effectors to proper (Golgi) membrane^11^.

Here we aimed to gain deeper structural understanding of complexes formed by PI4KB. These complexes are too large for NMR studies and also not suitable for characterization by protein crystallography because PI4KB itself and some of its interacting partners contain large intrinsically disordered regions. The lack of symmetry and conformational heterogeneity impedes also the cryoEM analysis. Because of these constrains we chose small angle X-ray scattering (SAXS) in combination with molecular simulations as a method suitable for large flexible protein complexes^42^.

## Results and Discussion

### Stoichiometry of PI4KB complexes

To correctly interpret SAXS data using molecular simulation based methods, the knowledge of stoichiometry of protein complexes is absolutely essential. Actually, even the interpretation of simple molecular envelopes (that can be very useful for rigid protein complexes) is also difficult without the knowledge of the exact stoichiometry. We have previously established that PI4KB and ACBD3 form a 1:1 complex^7^ and that 14-3-3 and PI4KB preferentially form 2:2 complex^32^. Previous crystallographic studies suggest that PI4KB:Rab11 form a 1:1 complex^17^ and the stoichiometry of 14-3-3:PI4KB:Rab11 protein complex was never experimentally tackled although based on the known stoichiometries of PI4KB:Rab11 (1:1) and 14-3-3:PI4KB (2:2) protein complexes we expected a 2:2:2 14-3-3:PI4KB:Rab11 complex to be formed.

We used analytical ultracentrifugation (AUC) to experimentally establish stoichiomentries of the aforementioned PI4KB complexes in solution. AUC revealed that PI4KB:Rab11 form a 1:1 complex as expected. PI4KB at the 20 µM concentration forms a dimer that disappears when Rab11 is added (Fig. 1B) probably because Rab11 sterically obstructs the dimerization although we cannot rule out other mechanisms because the dimerization interface is not known.

Next, we analyzed the 14-3-3:PI4KB:Rab11 protein complex by AUC. These experiments clearly show that the stoichiometry of this protein complex is 2:1:1 (Fig. 1C), meaning that one 14-3-3 dimer binds one molecule of phosphorylated PI4KB that, in turn, binds one molecule of Rab11. These stoichiometries are independent of Rab11 nucleotide state (SI Fig. 1).

### SAXS and modeling of the 14-3-3:PI4KB:Rab11 complex in solution

**Figure 2:**
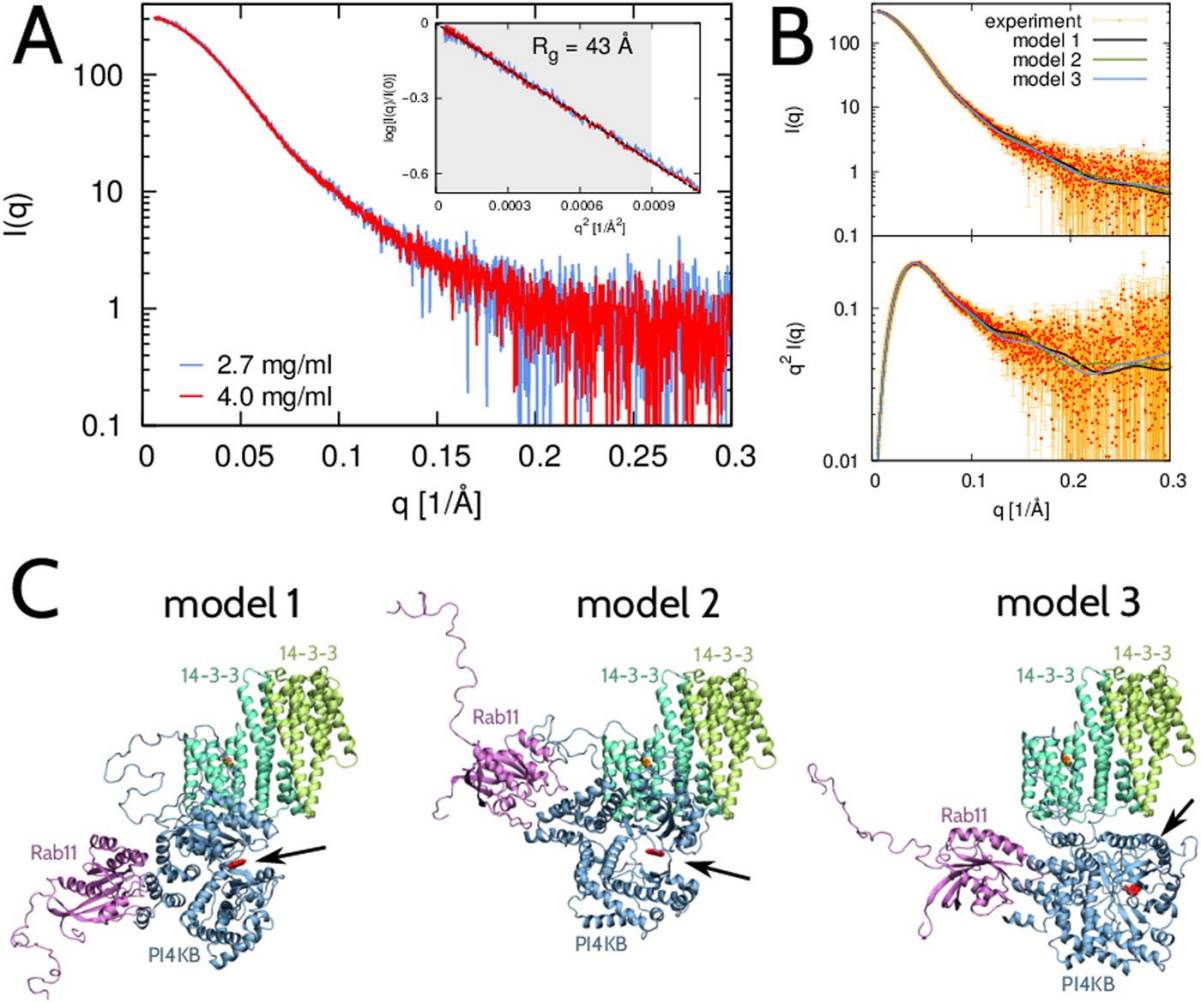
SAXS analysis and modeling of the 14-3-3:PI4KB:Rab11 protein complex in 2:1:1 stoichiometry. (**A**) Experimental SAXS intensity obtained at protein concentrations c = 2.7 mg/ml (blue) and c = 4.0 mg/ml (red). The logarithmic scale is used on the vertical axis. Inset: Guinier plot, i.e., log(I(q)/I(0)) vs. q^2^. The region qR_g_ < 1.3 where the Guinier approximation is valid for a globular protein is shaded gray. The dashed line indicates the best fit of the Guinier approximation, which leads to R_g_ = 43 Å. (**B**) Experimental (red) and theoretical (model 1 – black, model 2 – green, model 3 – blue) scattering data shown as I(q) vs. q (top) and q^2^I(q) vs. q (Kratky plot; bottom). Any of the three models fits the experimental SAXS data with χ^2^ = 1.2. (**C**) Three representative models of the 14-3-3:PI4KB:Rab11 complex (14-3-3 in green, PI4KB in blue, Rab11 in magenta) that fit the experimental SAXS data. Their scattering intensity profiles are shown in panel B in black, green and blue, respectively. The active site in PI4KB is indicated by arrows and highlighted in red.

We collected SAXS data on the 14-3-3:PI4KB:Rab11 ternary complex at protein concentrations of 2.7 and 4.0 mg/ml, which correspond to molar concentrations much larger than the dissociation constants of the 14-3-3:PI4KB and PI4KB:Rab11 protein complexes. The two datasets overlay after rescaling, indicating no protein aggregation in the samples (Fig. 2A). The Guinier plot (inset in Fig. 2A) shows that the radius of gyration of the 14-3-3:PI4KB:Rab11 protein complex is 43 Å suggesting a rather compact conformation.

Next, we used molecular simulations to structurally interpret the SAXS data and, hence, to identify conformations of the 14-3-3:PI4KB:Rab11 protein complex in solution. To this end, we simulated the 14-3-3:PI4KB:Rab11 complex in 2:1:1 stoichiometry, and identified a representative group of simulated structures consistent with the SAXS data (Fig. 2B). Similarly as in the case of the 14-3-3:PI4KB protein complex^32^, the 14-3-3:PI4KB:Rab11 protein complex appears to be rather compact (Fig. 2C), despite the long disordered region in PI4KB. The position of PI4KB in reference to the 14-3-3 dimer is different in 14-3-3:PI4KB:Rab11 than in 14-3-3:PI4KB but the active site of PI4KB is exposed in either case. The C-terminal tail of Rab11 is disordered. It attains extended conformations and never makes direct contacts with protein 14-3-3 in the ternary complex, which suggests that 14-3-3 should not interfere with the attachment of Rab11 to the membrane via its geranylgeranylated C-termini.

### SAXS and modeling of the ACBD3:PI4KB complex in solution

**Figure 3:**
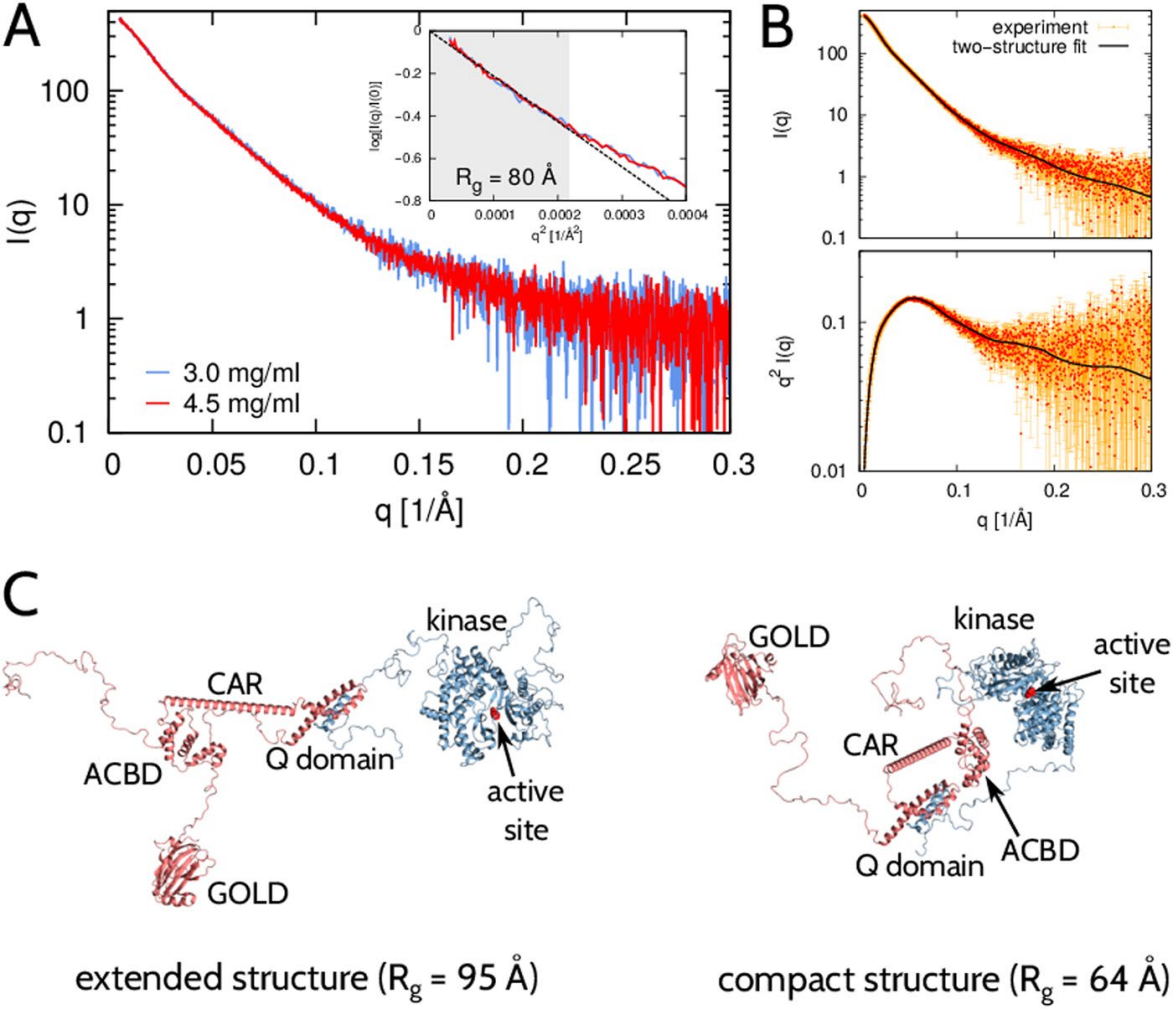
SAXS analysis and modeling of the ACBD3:PI4KB protein complex. (**A**) Experimental SAXS intensity obtained at protein concentrations c = 3.0 mg/ml (blue) and c = 4.5 mg/ml (red). Inset: Guinier plot, i.e., log(I(q)/I(0)) vs. q^2^. The region qR_g_ < 1.3 where the Guinier approximation is valid for a globular protein is shaded gray. The dashed line indicates the best fit of the Guinier approximation, which leads to R_g_ = 80 Å. (**B**) Experimental (red) and theoretical (black) scattering data shown as I(q) vs. q (top) and q^2^I(q) vs. q (Kratky plot; bottom). The theoretical curve corresponds to a minimal conformational ensemble that best fits the experimental SAXS data. (**C**) Two structural models of the ACBD3:PI4KB complex (ACBD3 in red, PI4KB in blue) that constitute the minimal conformational ensemble and jointly fit the experimental SAXS data with χ^2^ = 1.3. The protein domains of ACBD3 and PI4KB are indicated. The active site in PI4KB is highlighted by a modeled inhibitor (red) and indicated by arrows.

We performed SAXS measurements on the ACBD3:PI4KB protein complex at protein concentrations 3.0 and 4.5 mg/ml, which is about two orders of magnitude larger than the dissociation constant of the ACBD3:PI4KB complex (concentration of each protein ~10 µM and K_d_ ~ 300 nM). These two datasets overlay after rescaling, indicating no protein aggregation in the samples (Fig. 3A). The Guinier plot (inset in Fig. 3A) shows that the radius of gyration of the ACBD3:PI4KB protein complex is about 80 Å, suggesting that ACBD3:PI4KB can attain extended conformations in solution.

We next performed molecular simulations of the ACBD3:PI4KB protein complex and quantitatively compared the simulated SAXS curves to the experimental SAXS data. In contrast to the case of the 14-3-3:PI4KB:Rab11 complex, here we could not account for the ACBD3:PI4KB SAXS data with a single structure obtained from the simulations. However, we found that two structures together – an extended one and a more compact one – produced a scattering curve that was fully consistent with the SAXS data (Fig. 3B). These two structures (Fig. 3C) constitute thus a minimal representation of the ensemble of ACBD3:PI4KB conformations that fits the SAXS data. In each of the two structures, the inter-domain linker peptides are seen to be in extended conformations. However, in the compact structure, the ACBD domain, the Q domain and the CAR domain of ACBD3 are in proximity of the kinase domain of PI4KB. In contrast, in the extended structure, these domains are positioned far apart one another. In both of these two structures, the active site of PI4KB is exposed to solution, which explains why ACBD3 binding has no inhibitory effect on PI4KB^7^.

Since the full-length ACBD3:PI4KB protein complex contains several flexible loops and disordered linkers, it can attain multiple conformations, ranging from extended to compact ones. The two structures identified in our SAXS analysis (Fig. 3C) cannot faithfully represent the actual ensemble of possible conformations of the ACBD3:PI4KB protein complex in solution. However, the vast differences between the two structures provide a glimpse of the conformational heterogeneity of this flexible protein complex.

### Simulations of the full-length ACBD3:PI4KB:Rab11 complex at a lipid membrane

We also performed simulations of the ACBD3:PI4KB:Rab11 ternary complex at a lipid membrane. In these simulations, Rab11 was anchored to the lipid membrane by two geranylgeranylated Cys residues at its C-terminus. Moreover, ACBD3 was held at the membrane at two sites – one site was on the ACBD domain (which binds acyl-CoA) and the other one was on the GOLD domain – see Materials and Methods for details. In addition to these three sites at which ACBD3:PI4KB:Rab11 was anchored to the membrane, our simulations revealed several other protein sites interacting transiently with the lipid membrane. Among these sites were positively charged segments of the disordered region between Met223 and Asn314 in PI4KB. (We note that this long region is missing in crystal structures of PI4KB). We speculate that these segments, which are enriched in Arg and Lys residues, may help to position PI4KB on the membrane and could explain why the full length PI4KB is about 30% more active compared to the loop deletion mutant^43^.

**Figure 4:**
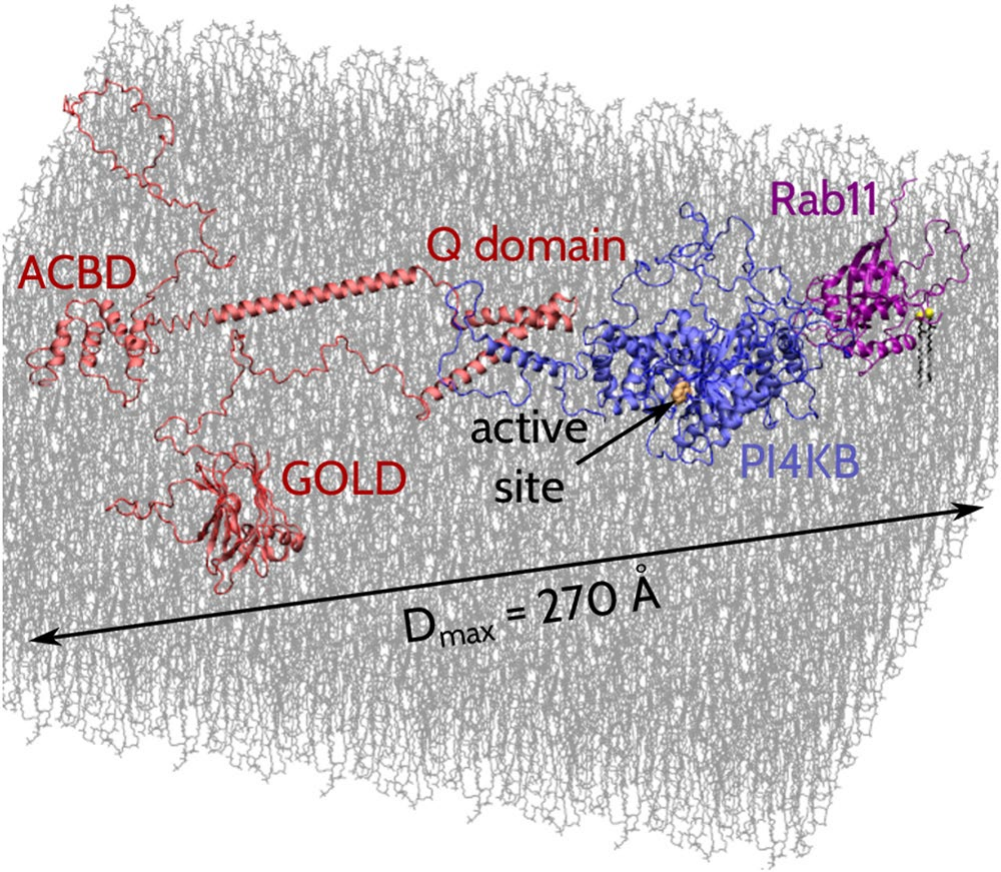
A simulation snapshot of the ACBD3:PI4KB:Rab11 protein complex at a lipid bilayer. The maximum extension of ACBD3:PI4KB:Rab11 is 270 Å in this conformation. ACBD3 is shown in red, PI4KB in blue, Rab11 in violet, and lipid bilayer in gray. The individual domains of ACBD3 are labeled in red. The active site of PI4KB is marked by an arrow. The two geranylgeranylated Cys residues at the C-terminus of Rab11 are shown in the stick representation.

In our simulations, the maximum extension *D*_max_ of the ACBD3:PI4KB:Rab11 ternary complex at the membrane was found to vary approximately between 220 Å and 340 Å with the average of about 275 Å (Fig. 4). This observation indicates that ACBD3:PI4KB:Rab11 exhibits large conformational fluctuations while being bound to the membrane. This finding explains why membrane curvature dependent activity of PI4KB was never reported when it was reported for other lipid kinases^44^.

### Membrane assembly of PI4KB complexes

Next, we aimed to verify our structural prediction that PI4KB recruited to the membrane by ACBD3 would be able to recruit Rab11. We decided to work at the physiologically relevant nanomolar concentrations because at these protein concentrations PI4KB complexes assemble in cells. First we used the GUV biomimetic system where ACBD3 was tethered to the GUV membrane and was able to recruit PI4KB as previously reported by us and others^7,33,36^. However, when Rab11 was added (at the 50 nM concentration) we did not observe any Rab11 recruitment to the membrane surface (SI Fig. 2A). This is perhaps not surprising because the published K_d_ of Rab11 and PI4KB is between 4 to 20 µM depending whether Rab11 is GTP or GDP bound^17^. However, PI4KB was reported to recruit Rab11 in cells^11^.

**Figure 5:**
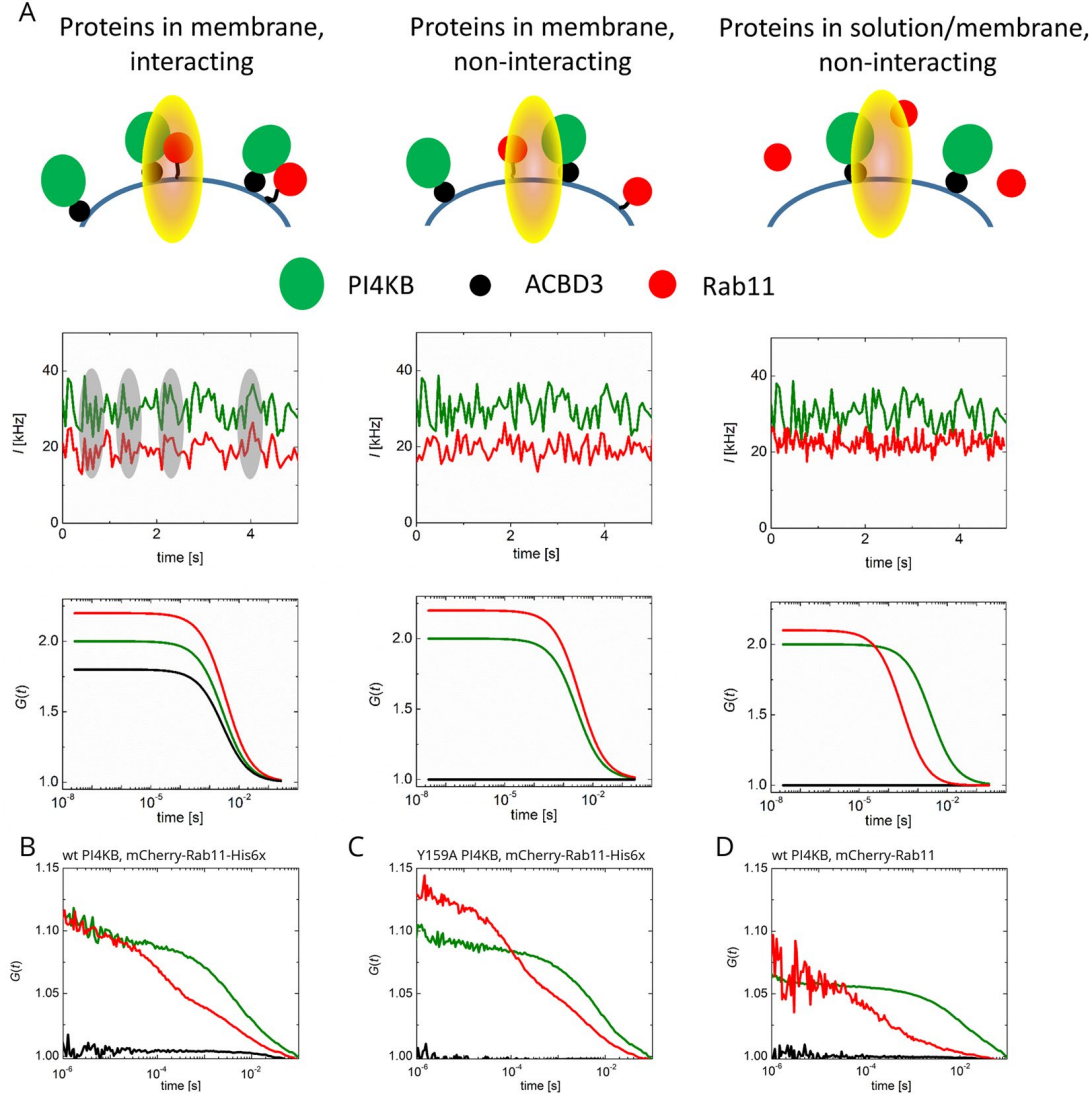
Reconstitution of the ACBD3:PI4KB:Rab11 protein complex at the lipid bilayer. (**A**) Scheme of the microscopy experiment and illustrative readouts Upper panel: scheme of the PI4KB complex on the GUV membrane. PI4KB (green) is attached to the membrane by ACBD3 (black); mCherry-Rab11 (red) is either membrane tethered via His-Tag, or stays in solution above the membrane. The interaction is visualized by joint motion of the proteins within the laser illuminated area. Middle panel: Joint motion of the fluorescence labeled proteins manifests itself by simultaneous fluctuations of the fluorescence intensity in green and red detection channel (marked in grey). If proteins do not move together, i.e. do not interact, the green/red fluctuations are uncorrelated. Bottom part: Temporal autocorrelation functions (ACFs) of the fluorescence intensity in green and red channel (green and red lines) refer on the diffusion rate (full width in half maximum) and concentration (amplitude) of individual proteins. The amplitude of the cross-correlation functions (CCFs, black) quantifies the pair-wise motion of the proteins. (**B**) wild type PI4KB and membrane tethered mCherry-Rab11-His; (**C**) Y159A PI4KB and membrane tethered mCherry-Rab11-His; (**D**) wild type PI4KB and soluble mCherry-Rab11.

Rab11 is stably associated with membranes in cells via its geranylgeranylated C-termini. We hypothesized that membrane association of Rab11 is essential for the formation of ACBD3:PI4KB:Rab11 protein complex at physiologically relevant protein concentrations. To test this hypothesis, we could not use the simple colocalization assay because membrane tethered Rab11 will be always localized on the surface of the membrane (SI Fig. 2B). Instead we used cross-correlation spectroscopy to directly observe correlation between fluorescence signals of Atto488 labeled PI4KB and mCherry-Rab11. The cross-correlating signal refers on the joint motion of the two proteins and thus directly reports on their interaction (Fig. 5A). We observed the formation of the ACBD3:PI4KB:Rab11 complex when membrane tethered Rab11 was used (Fig. 5B). The amplitude of the cross-correlation function was relatively low (especially when compared to the ideal case as in Fig. 5A) suggesting that only ~10% of Rab11 was localized in the complex, however, the effect was specific - when we used Y159A PI4KB mutant that does not bind Rab11^17^ we did not observe any ACBD3:PI4KB:Rab11 complex (Fig. 5C) which is manifested by the flat cross-correlation curve that only oscillates around the ground level as a result of noise. We could not increase the protein concentration because of the single-molecule nature of the FCCS experiment. Despite the low protein concentrations used the difference between wild type and Y159A mutant PI4KB are statistically significant at the 95% level of probability (SI Table 2). We also didn’t observe any ACBD3:PI4KB:Rab11 complex formation when soluble Rab11 was used (Fig. 5D) just as previously in the GUV recruitment assay (SI Fig. 2). These results indicate that the membrane plays an important role and is in fact indispensable for the formation of ACBD3:PI4KB:Rab11 protein complex and thus for the recruitment of Rab11 and its effectors.

## Concluding Remarks

Crystallographic and SAXS analysis coupled to molecular simulations shed some light on the behavior of large flexible complexes in solution and even provided snapshots of their actions on the membrane^45–49^. Here we described the conformations of several PI4KB complexes in solution. We used molecular simulations to gain insights into the conformational flexibility on the membrane surface. Our analysis revealed that ACBD3:PI4KB:Rab11 protein complex is extremely flexible and that the membrane is important for its formation. The observed effect is probably due to high local concentration. When membrane tethered proteins are used their local concentration on the membrane surface (or 2D concentration) can be high despite the average concentration (or 3D concentration) being in the nanomolar range. We believe that the cellular Golgi membrane acts in a similar manner when the Golgi localized ACBD3 recruits the PI4KB to the Golgi facilitating high local concentration of PI4KB that is subsequently responsible for the recruitment of Rab11 and its effectors to the Golgi.

## Materials and Methods

### Protein expression and purification

All recombinant proteins were expressed in *E*. *coli* strain BL21 NiCo in an auto induction media 16 h in 18 °C using our standard protocols^50,51^. Briefly, cells expressing the proteins were centrifuged, lysed in 50 mM Tris pH 8, 20 mM Imidazole, 300 mM NaCl, 10% glycerol, and 3 mM β-mercaptoethanol (βME) using EmulsiFlex-C3 homogenizer, lysate was centrifuged at 30,000 × g for 20 min at 4 °C and the supernatant was further used for purification of the proteins.

14-3-3 was expressed as a fusion protein with N-terminal 6xHis tag followed by TEV protease site. Protein was purified from the lysate using affinity chromatography on Ni-NTA resin (Macherey-Nagel) and the His tag was then cleaved off by TEV protease. Size-exclusion chromatography (SEC) on Superdex 75 HiLoad 16/60 column (GE Healthcare) in 30 mM Tris pH 8, 200 mM NaCl, and 3 mM βME was used as the next purification step. Purified protein was concentrated to 20 mg/ml and flash-frozen in liquid nitrogen.

Rab11 was also expressed as a fusion protein with N-terminal 6xHis tag followed by TEV cleavage site and purified using affinity chromatography and TEV cleavage. Next, Rab11 was dialyzed against 30 mM Tris pH 8, 200 mM NaCl, 3 mM βME, and 5 mM EDTA (to dissociate GDP), and further purified by SEC on Superdex 75 HiLoad 16/60 column in 30 mM Tris pH 8, 200 mM NaCl, and 3 mM βME. Finally, Rab11 was concentrated to 20 mg/ml and flash-frozen in liquid nitrogen.

Rab11 used for membrane assembly experiments was expressed as fusion protein with N-terminal 6xHis tag followed by TEV cleavage site and mCherry on its N-terminus and four amino acid linker (SGTG) followed by 8xHis tag on its C-terminus. This protein was further purified the same way as Rab11 used for SAXS experiments. (After cleavage of the N-terminal 6xHis tag by TEV protease the purified protein still contained mCherry and C-terminal 8xHis tag).

PI4KB must be phosphorylated at S294 in order to interact with 14-3-3 protein. We used the same approach as previously^32^. Briefly, construct of PI4KB residues 128-799 with internal deletion of a disordered loop (residues 423-522)^17^ to facilitate bacterial expression and protein stability and single point mutation T292R was used to faciliate *in vitro* phosphorylation by protein kinase A. The protein was expressed as a fusion protein with 8xHis-SUMO solubility tag and purified using affinity chromatography followed by the SUMO tag cleavage (by the Ulp1 enzyme from *S*. *cerevisiae*). Next, we performed SEC on Superdex 200 HiLoad 16/60 column in 30 mM Tris pH 8, 200 mM NaCl, and 3 mM βME. Purified PI4KB was then phosphorylated by PKA (4 μg of PKA per 1 mg of PI4KB were incubated in 20 mM Tris pH 7.4, 150 mM NaCl, 2 mM DTT, 300 μM ATP, and 10 mM MgCl_2_ for 2 h at 25 °C and subsequently for 4 h at 4 °C). Phosphorylated PI4KB was further purified by anion-exchange chromatography on Mono Q 5/50GL (GE Healthcare) and by SEC on Superdex 200 HiLoad 16/60 column in 20 mM Tris pH 8, 200 mM NaCl, and 3 mM βME. Finally, it was concentrated to 12 mg/ml and flash-frozen in liquid nitrogen.

PI4KB must contain its intact N-terminus in order to interact with the ACBD3 protein, therefore we used a construct described previously that contains only the internal deletion and has a 6xHis- GB1 solubility tag followed by TEV protease site that is further on referred to as pseudo-wt PI4KB^7^. Pseudo-wt PI4KB was also purified by affinity chromatography followed by TEV protease cleavage and SEC (Superdex 200 HiLoad 16/60 column in 30 mM Tris pH8, 200 mM NaCl, and 3 mM βME) and by anion exchange on Mono Q 5/50GL. Purified protein was dialyzed against 20 mM Tris pH 8, 200 mM NaCl, 2 mM TCEP, and 1% glycerol, concentrated to 5 mg/ml and flash-frozen in liquid nitrogen.

Pseudo-wt PI4KB used for membrane assembly experiments was labeled with Atto488 maleimide (Sigma-Aldrich) according to the manufacturer’s protocol. The labeled protein was purified from unbound dye on HiTrap Desalting column (GE Healthcare) in 20 mM Tris pH 8, 200 mM NaCl, 3 mM βME, and concentrated to 1 mg/ml. Pseudo-wt PI4KB Y159A (mutation Y159A prevents pseudo-wt PI4KB from binding Rab11^17^) used as negative control in membrane assembly experiments was purified and labeled in the same way.

ACBD3 with N-terminal 6xHis-GB1 solubility tag was purified by affinity chromatography followed by TEV cleavage (except for protein for GUV reconstitution assays) and by SEC (Superdex 200 HiLoad 16/60 column in 20 mM Tris pH 8, 200 mM NaCl, and 3 mM βME). Purified ACBD3 was concentrated to 6 mg/ml and flash-frozen in liquid nitrogen.

### Analytical ultracentrifugation analysis (AUC)

AUC experiments were performed in the sedimentation velocity mode using the ProteomeLab XL-I Beckman Coulter analytical ultracentrifuge. All measurements were conducted at several loading concentrations and molar ratios as indicated in the corresponding figures in charcoal-filled Epon centerpieces with 12-mm optical path length in the size-exclusion chromatography buffer at 20 °C and 42 000 rpm. The buffer density, viscosity, and partial specific volume of all proteins were estimated using the program SEDNTERP (http://sed-nterp.unh.edu/). All data were collected with interference and/or absorbance optics at 280 nm and analyzed in terms of a continuous c(s) distribution of Lamm equation solutions with the software SEDFIT^52^.

### Small angle X-ray scattering (SAXS) measurements

Sample of the ACBD3:PI4KB complex was prepared by mixing the recombinant proteins ACBD3 and pseudo-wt PI4KB in 1:1 molar ratio, then dialyzed against 20 mM Tris pH 8, 200 mM NaCl, 2 mM TCEP, and 1% glycerol. The complex was then concentrated to 3 and 4.5 mg/ml and flash-frozen in liquid nitrogen.

Sample of the complex 14-3-3:PI4KB:Rab11 was prepared by mixing 14-3-3, phosphorylated pseudo-wt PI4KB, and Rab11 in molar ratio 1:1:1 and further purified on Superdex 200 10/300GL in 20 mM Tris pH 8, 150 mM NaCl, 1 mM TCEP, and 1% glycerol. Then the complex was concentrated to 2.7 and 4 mg/ml and flash-frozen in liquid nitrogen.

The SAXS data were collected using beamlines BioSAXS Beamline BM29 (ESRF Grenoble) and EMBL SAXS beamline P12 (Petra III DESY, Hamburg) that are both equipped with the 2 M Pilatus detector (Dectris).

### Modeling and simulations of the full-length 14-3-3:PI4KB:Rab11 complex in solution

To efficiently sample conformations of the full-length 14-3-3:PI4KB:Rab11 ternary complex in solution, we used coarse-grained simulations in which the crystallized portions of proteins were treated as rigid bodies whereas the flexible loops and disordered segments were simulated as chains of amino-acid beads with appropriate bending, stretching and torsional potentials^53^. Here, the crystallized portion of the PI4KB:Rab11 complex with the PDB code 4D0L^17^ was taken as one rigid body. The structure of the 14-3-3 dimer in complex with the PI4KB-derived phosphopeptide (PDB code: 5NAS)^54^ was used as another rigid body, as in our previous computational study of the 14-3-3:PI4KB protein complex^32^. Within this approach, two independent Monte Carlo (MC) simulation runs of the 14-3-3:PI4KB:Rab11 complex in the 2:1:1 stoichiometry were performed. To enhance sampling and generate a pool of diverse structures for SAXS analysis, the replica exchange (RE) method was adopted with replicas at 16 different temperatures ranging from 300 to 500 K. The two REMC simulation runs were started from different conformations of the protein complex. Each of the simulations involved 5·10^6^ MC sweeps. The simulation structures were saved every 1000 MC sweeps. In this way, we obtained 2·16 = 32 trajectories, with 5000 simulation structures recorded in each.

The resulting pool of structures was very diverse. The simulation structures had the radius of gyration, R_g_, ranging from 37 to 59 Å. Their maximum extension, D_max_, was found to vary between 110 and 205 Å. The scattering intensity profile was computed for each of the simulation structures individually using the algorithm co-developed with the EROS method^55^. The discrepancy between the experimental SAXS data, I_exp_(q), and the scattering intensity profile of the *k*-th simulation structure, I_k_(q), was quantified according to equation 1:

1 $${\chi }_{k}^{2}=\sum _{i=1}^{{N}_{q}}\frac{{({I}_{\exp }({q}_{i})-a{I}_{k}({q}_{i}))}^{2}}{{\sigma }^{2}({q}_{i})}$$

Here, the index *k* labels the simulation structures, N_q_ is the number of SAXS data points, σ(q) is the statistical error of the measured SAXS intensity, I_exp_(q), whereas the parameter *a* sets the intensity scale and is obtained from the condition ∂χ^2^/∂*a* = 0.

Out of the pool of 32·5000 = 160,000 simulation structures, we selected 100 structures with the smallest values of χ^2^, which were found to be within the range from 1.1 to 1.2. The top 100 structures have the radius of gyration in the range between 42.2 and 44.2 Å with the average at about 43 Å. Their maximum extension is spread in the range between about 145 and 190 Å with the average at 162 Å. In terms of their characteristic sizes, R_g_ and D_max_, these models are quite typical within the pool of the simulation structures.

We sorted the top 100 structures according to their similarity in the position of the crystallized portion of the PI4KB:Rab11 complex relative to the 14-3-3 dimer. We used the QT-clustering method with DRMS as metric^56^. We obtained one major cluster and three or four minor clusters, depending on the cutoff on DRMS. For example, for the DRMS cutoff of 5 Å, the major cluster contains 52 structures, and the two minor clusters contain 18 and 17 structures. The simulation structures representing these three clusters are shown in Fig. 2C.

### Modeling and simulations of the full-length ACBD3:PI4KB complex in solution

We performed analogous REMC simulations of the ACBD3:PI4KB protein complex. The simulation model of ACBD3:PI4KB was composed of five rigid domains: (i) the crystallized portion of PI4KB with the PDB code 4WAE^20^, which contains amino-acid residues with numbers ranging from 130 to 423 and from 522 to 816; (ii) a homology model of the ACBD domain (residue numbers from 82 to 179) based on the PDB entry 3FLV (29% sequence identity, 96% sequence coverage); (iii) the ACBD3 charged amino-acid region (CAR, residue numbers from 180 to 240) represented by a single alpha-helix, as proposed by Suveges *et al*.^57^; (iv) the ACBD3 glutamine-rich domain (Q domain, residue numbers from 241 to 308) in complex with a N-terminal α-helix of PI4KB (residue numbers from 40 to 63), as given by the NMR structure with the PDB code 2N73^7^; and (v) the crystallized portion of the ACBD3 Golgi dynamics domain (GOLD domain) with the PDB code 5LZ1 (residue numbers ranging from 128 to 422 and from 523 to 799)^36^. All of the missing loops, disordered linkers and terminal segments were modeled using ModLoop^58^ and simulated as chains of amino-acid beads with appropriate bending, stretching and torsional potentials.

The REMC simulations provided a very diverse pool of 80,000 structures with R_g_ varying from 40 to 110 Å and D_max_ in the range between 130 and 360 Å. Curiously, none of the simulation structures from this pool could be fit to the experimental SAXS data with χ^2^ < 1.8. Since the full-length ACBD3:PI4KB complex contains numerous flexible loops and disordered linkers, we expected it to exhibit conformational diversity and flexibility in solution. Therefore, we applied a minimum-ensemble method^59^ to gain structural interpretation of the SAXS data. The minimum ensemble consistent with the SAXS data was found to be represented by two simulation structures, i.e., one ‘extended’ with R_g_ = 95 Å and D_max_ = 300 Å, and one ‘compact’ with R_g_ = 64 Å and D_max_ = 200 Å (Fig. 3C). These two structures taken together with equal statistical weights fit the experimental SAXS data with χ^2^ = 1.3 (Fig. 3B).

### Modeling and simulations of the full-length ACBD3:PI4KB:Rab11 ternary complex at a lipid membrane

To sample conformations of the ACBD3:PI4KB:Rab11 ternary complex at a bilayer membrane, we used the same coarse-grained simulation package as for the simulations of ACBD3:PI4KB and 14-3-3:PI4KB:Rab11 in solution. In the framework of this model, the interactions of the residue beads with the membrane are described by statistical amino-acid dependent potentials and Debye-Hückel-type electrostatics. A detailed description of this approach can be found in Kim *et al*.^53^.

Rab11 is geranylgeranylated at two Cys residues in its C-terminus. We simulated this lipid modification in an analogous way as the palmitoylation of four Cys residues in PI4K IIα in our earlier computational studies^18^. Namely, to mimic the anchoring of the two geranylgeranylated Cys residues in the lipid membrane, we imposed soft harmonic potentials on the *z*-coordinates of these Cys residue beads relative to the membrane surface located at *z* = 2 nm.

ACBD3 is held at the membrane at two sites that are located on two separate domains. Namely, the ACBD domain binds acyl-CoA whereas the GOLD domain has a membrane binding site that has been identified in our earlier studies on the GOLD:3 A complex^36^. In our simulations, ACBD3 was thus restrained to the membrane surface, positioned at *z* = 2 nm, by soft harmonic potentials acting on the *z*-coordinates of these two sites in the ACBD and GOLD domains.

We performed 20 independent REMC simulations of ACBD3:PI4KB:Rab11 restrained to the flat membrane as described above. Each of the simulations was started from a different ACBD3:PI4KB:Rab11 conformation and with a different seed for the random number generator. Each of the simulations involved replicas at 8 different temperatures and 2.5·10^6^ MC sweeps. The simulation structures were saved every 1000 MC sweeps at room temperature only. In this way, we obtained an ensemble of 20·2500 = 50,000 coarse-grained structures for further analysis.

For each of the recorded ACBD3:PI4KB:Rab11 conformations we identified residue beads with the *z*-coordinate below a threshold *z* = *z*_0_. We investigated the values of *z*_0_ in the range between 2 and 2.5 nm. In this way, we estimated the probabilities of contacts between individual residue beads and the flat membrane. We also analyzed the maximum extension *D*_*max*_ of the ACBD3:PI4KB:Rab11 protein complex at the membrane.

### Giant unilamellar vesicles (GUVs) preparation and imaging

GUVs were prepared as before^60^, briefly, chloroform lipid mixture containing 55 mol % of POPC, 10 mol % POPS, 10 mol % PI, 20 mol % cholesterol, and 5 mol % DGS-NTA(Ni) (all Avanti Polar Lipids) was prepared at overall lipid concentration 5 µg/µL. 10 µL of the lipid mixture was spread on two ITO coated glass electrodes each. The electrodes were dried under vacuum overnight and then parallel assembled into a home made teflon chamber containing 5 mL of 600 mM sucrose solution. For the electroformation, 10 Hz, harmonically oscillating voltage of 1 V peak value was applied to the electrodes for 1 hour at 60 °C. For imaging, BSA-coated 4-chamber glass bottom dish (*In Vitro* Scientific) were used. 100 µL of GUVs mixed with 100 µL isosmotic buffer (25 mM Tris pH 8, 10 mM MgCl_2_, 20 mM Imidazole, 261.5 mM NaCl, 2 mM βME) containing proteins of interest, so that the final concentrations of the proteins (ACBD3, PI4KB, mCherry-Rab11) were approximately 50 nM.

The images of GUVs were acquired on LSM 780 confocal microscope (Zeiss, Jena, Germany) using 40x/1.2 water objective. The images were taken line-sequentially in two tracks: Atto488 and mCherry (excitation/emission wavelengths: 490 nm/499–525 nm, and 561 nm/577–640 nm, respectively).

For fluorescence correlation spectroscopy (FCS) experiments the LSM 780 was equipped with external tau-SPAD detectors and time-correlated single photon counting (TCSPC) electronics (Hydraharp, Picoquant, Berlin, Germany). For the Atto488 excitation, 490 nm line of the Intune laser (Ziess) pulsing at 40 MHz repetition frequency was used. mCherry was excited continuously at 561 nm. The excitation light was focused by 40x/1.2 W objective (Zeiss) into the solution. Fluorescence intensity, collected by the same objective lens, was re-focused on the pinhole (1 airy unit) and the re-collimated light behind the pinhole was split on the external tau-SPADs in front of which emission band pass filters 525/45 and 600/52 for Atto488 and mCherry signal, respectively, were placed.

The collected data were correlated by home-written script in Matlab (Mathworks, Natick, MA) according to the previously described algorithm^61^. To avoid detector crosstalk, the red channel fluorescence signal was split according to its TCSPC pattern (exponential for the signal generated by the pulsed Intune laser and flat for the 561 nm continuous wave laser) into two contributions and only the signal assigned to the flat TCSPC profile was correlated as described in detail previously^62^.

## Supplementary information

Supplementary information
