# Supplementary material for: Phosphatidylinositol 4-kinase IIIβ (PI4KB) forms highly flexible heterocomplexes that include ACBD3, 14-3-3, and Rab11 proteins
Source: Sci Rep. 2019 Jan 24;9:567. doi: 10.1038/s41598-018-37158-6 (PMC6345845; doi:10.1038/s41598-018-37158-6)
Supplement: Supplementary file 1 — Supplementary information [file 41598_2018_37158_MOESM1_ESM.docx]

**Supplementary Data**

**Phosphatidylinositol 4-kinase IIIβ (PI4KB) forms highly flexible heterocomplexes that include ACBD3, 14-3-3, and Rab11 proteins**

Dominika Chalupska^1^, Bartosz Różycki^2^, Jana Humpolickova^1^, Lenka Faltova^3^, Martin Klima^1^, and Evzen Boura^1^

^1^Institute of Organic Chemistry and Biochemistry of the Czech Academy of Sciences, Flemingovo nam. 2., 166 10 Prague 6, Czech Republic

^2^Institute of Physics, Polish Academy of Sciences, Al. Lotnikow 32/46, 02-668 Warsaw, Poland

^3^Laboratory of Biomolecular Research, Department of Biology and Chemistry, Paul Scherrer Institute, 5232, Villigen PSI, Switzerland

*correspondence to [boura@uochb.cas.cz](mailto:boura@uochb.cas.cz)

Running title: Structure of heteromeric PI4KB protein complexes

**Keywards**: lipid kinase; PI4KB; 14-3-3; ACBD3; Rab11; phosphatidylinositol; phosphatidylinositol 4-phosphate; structure; intrinsically disordered regions; small‐angle X‐ray scattering (SAXS); analytical ultracentrifugation (AUC); giant unilamellar vesicle (GUV)

**SI Figure**

**
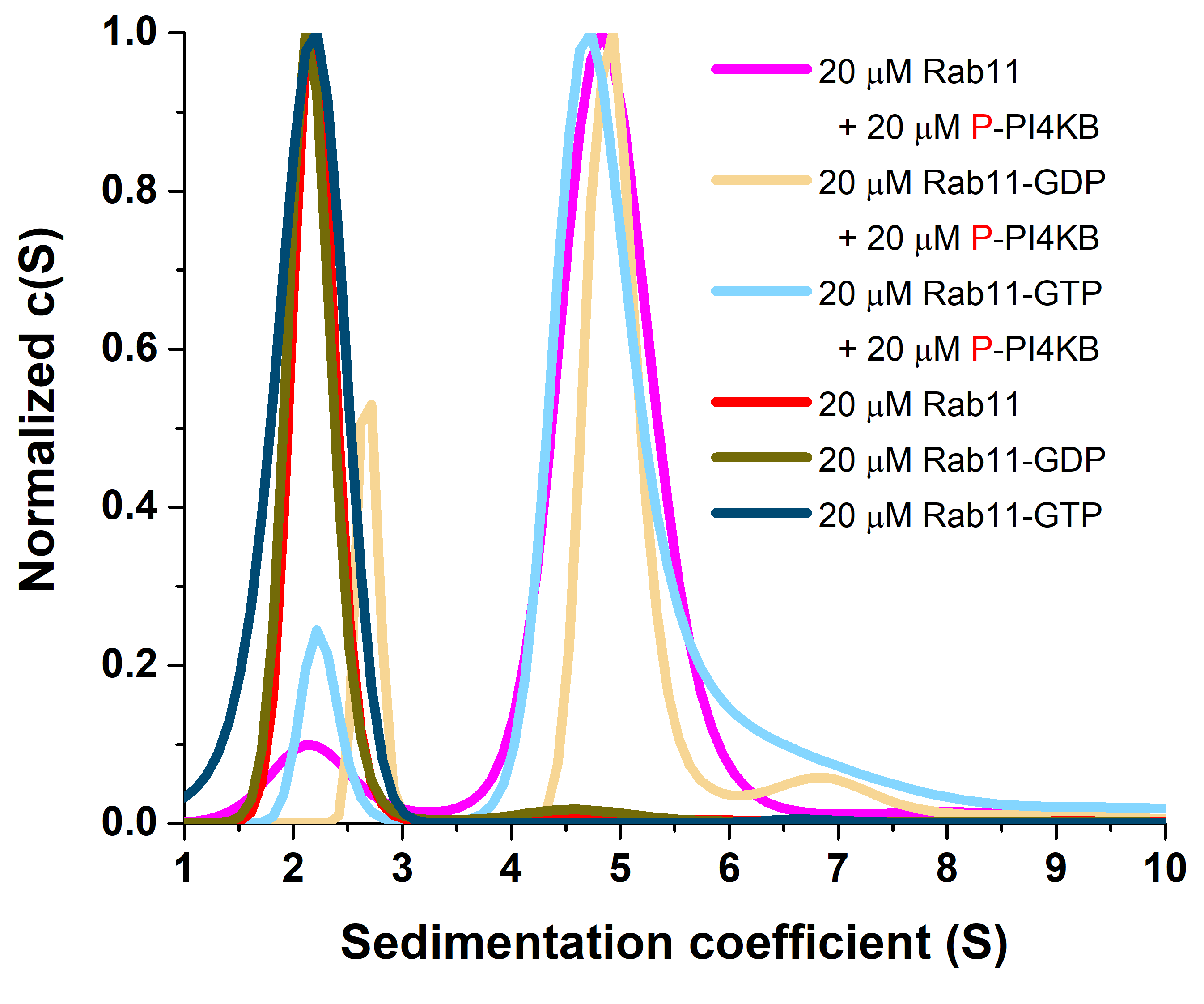
**

SI Fig. 1: **c (S) distribution profiles showing that 14-3-3:P-PI4KB:Rab11 2:1:1 stoichiometry is independent of Rab11 nucleotide state**


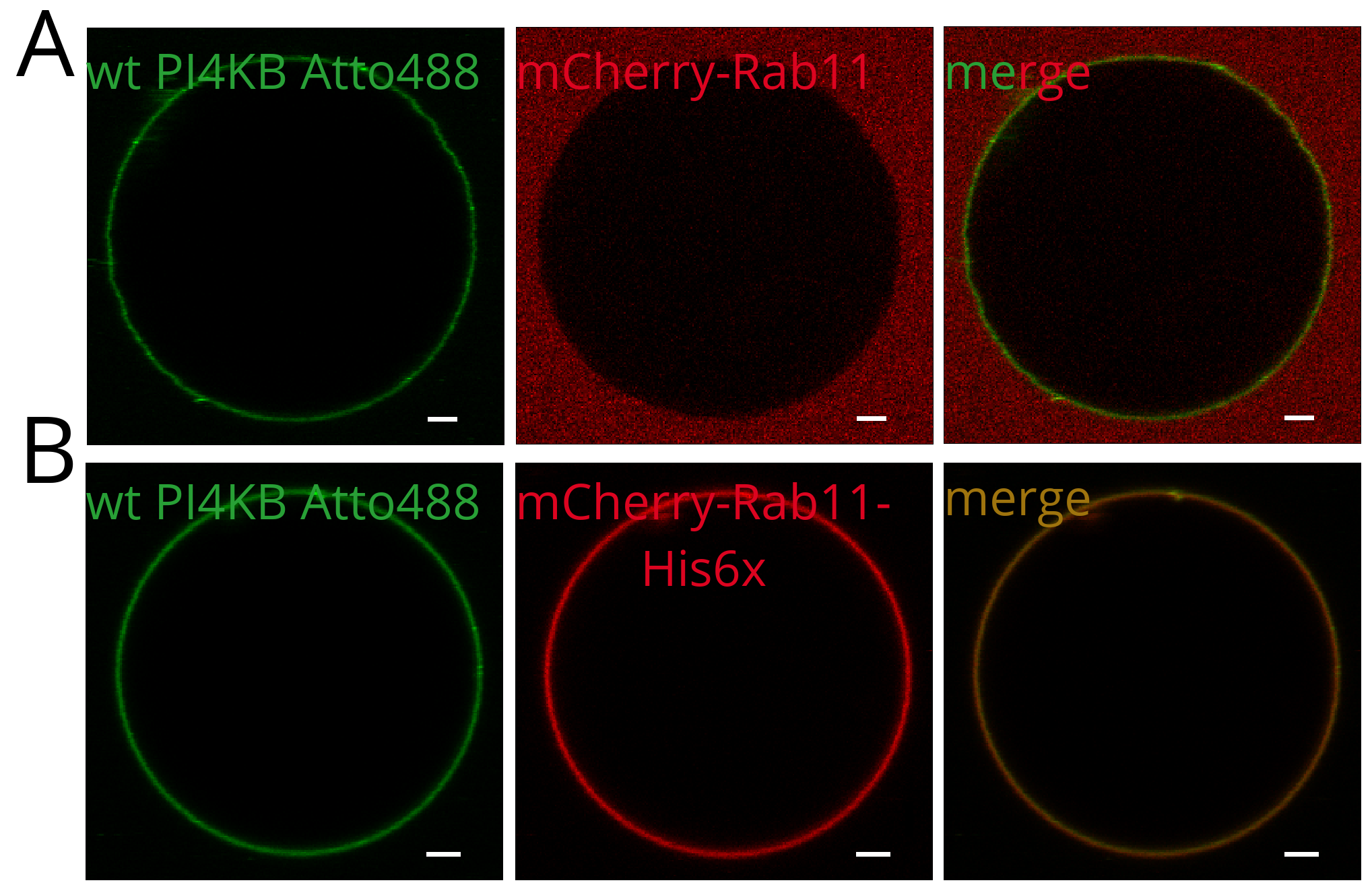


SI Fig. 2: **SI4KB does not recruit soluble Rab11 to membranes at physiological concentrations**: A) PI4KB-Atto488 membrane tethered by ACBD3 in green and mCherry-Rab11 in red B) PI4KB-Atto488 membrane tethered by ACBD3 in green, mCherry-Rab11-His6x in red. The membrane contains 5% of Ni-NTA lipid as detailed in the M&M section.

|  |  |  |  |
| --- | --- | --- | --- |
|  | protein | sw (main peak) |  |
|  | 20 µM 14-3-3 dimer | 3.5 |  |
|  | 20 µM Rab11 | 2.2 |  |
|  | 20 µM P-PI4KB | 5.3 |  |
|  | 20 µM P-PI4KB:20 µM 14-3-3 | 7.5 |  |
|  | 20 µM P-PI4KB: 20µM 14-3-3: 20 µM Rab11 | 6.7 |  |
|  | 20 µM P-PI4KB: 10µM 14-3-3: 10 µM Rab11 | 6.6 |  |
|  | 20 µM Rab11: 20 µM P-PI4KB | 4.8 |  |
|  | 20 µM Rab11: 10 µM P-PI4KB | 4.5 |  |
|  | 10 µM Rab11: 20 µM P-PI4KB | 5.1 |  |
|  |  |  |  |

SI Table 1: Sedimentation coefficients of all complexes analyzed by AUC

| PI4KB type | PI4KB-Atto488 | Rab11-mCherry | PI4KB-Rab11 | *K*_D_ |
| --- | --- | --- | --- | --- |
|  | Molecules/PSF | Molecules/PSF | Molecules/PSF | Molecules/PSF |
| Wt | 9.8 | 9.5 | 0.6 | 155 |
| Wt | 23.0 | 9.5 | 0.9 | 241 |
| Wt | 23.3 | 21.2 | 0.7 | 729 |
| Wt | 22.7 | 20.9 | 1.4 | 333 |
| Wt | 8.8 | 19.8 | 0.3 | 540 |
| Y159A | 15.3 | 7.1 | 0.1 | 939 |
| Y159A | 15.2 | 6.0 | 0.1 | 735 |
| Y159A | 13.0 | 9.8 | 0.2 | 685 |
| Y159A | 15.8 | 13.4 | 0.2 | 1117 |
| Y159A | 13.8 | 10.0 | 0.1 | 995 |

SI Table 2: Number of PI4KB-Atto488, Rab11-mCherry and PI4KB-Rab11 protein complexes within the laser illuminated spots (point spread function – PSF) obtained from fluorescence crosscorrelation spectroscopy (FCCS) experiment and corresponding value of the dissociation constant *K*_D_. According to the Student`s t-test, the two sets of *K*_D_s do not originate from the random distributions of the same mean at less than 5 % significance level.
